# Craniofacial morphology/phenotypes influence on mandibular range of movement in the design of a mandibular advancement device

**DOI:** 10.1186/s12903-020-01369-z

**Published:** 2021-01-07

**Authors:** P. Mayoral Sanz, M. Garcia Reyes, A. Bataller Torras, J. A. Cabrera Castillo, M. O. Lagravère Vich

**Affiliations:** 1 Master Program Dental Sleep Medicine, Catholic University of Murcia UCAM, Conde de Peñalver 61, 28006 Madrid, Spain; 2 Faculty of Engineering, University of Malaga, Flauta Mágica 22, 29006 Málaga, Spain; 3 Faculty of Medicine and Dentistry, School of Dentistry, University of Alberta ECHA, 5-524, 11405-87 Avenue, Edmonton, AB T6G 1C9 Canada

**Keywords:** Obstructive sleep apnea, Mandibular advancement device, Oral appliance design, Mandibular position, Vertical opening

## Abstract

**Background:**

The mandibular opening path movements have different directions according to the craniofacial morphology of the patient but always downward and backward, therefore increasing the collapse of the upper airway. The aim of this work is to determine if there is a relationship between the craniofacial morphology and the mandibular movement to help understand the impact on the mandibular position.

**Methods:**

52 students with full permanent dentition aged 19 to 23 years (mean 21.3 SD 1.7; 29 females and 23 males), participated in the study. Each subject had a lateral cephalometric radiograph taken. The opening angle was determined for two levels of vertical openings at 5 and 10 mm.

**Results:**

The opening angle showed a greater variability between subjects ranging from 63.15 to 77.08 for 5 mm angle and from for 61.65 to 75.72 for the 10 mm angle. Differences of facial phenotypes was evident when comparing the individual dissoccluding angle of the low angle horizontal pattern and high angle vertical pattern.

**Conclusions:**

The opening angle is related to craniofacial morphology with higher vertical anterior and shorter anteroposterior faces having a more horizontal path of mandibular movement than shorter vertical anterior and longer anteroposterior subjects who have a more vertical path.

## Background

Obstructive sleep apnea (OSA) is an important health problem, which has the presence of repeated episodes of a partial (hypopnea) or complete (apnea) collapse of the upper airway [1]. The consequences are fragmentation of sleep structure, a decrease in oxygen saturation and an increase in blood pressure [2]. The arousals and the nocturnal hypoxemia can lead to excessive daytime sleepiness, loss of concentration and hypertension [3, 4].

One treatment option for OSA is the use of a mandibular advancement device (MAD) [1, 5]. MADs keep the mandible in a protruded position during sleep increasing the width of the airway and reducing its collapsibility [6]. Even though there are conflicting reports on the success rate of these appliances [1, 7], MADs have been reported to be an alternative treatment to CPAP in moderate to severe OSA cases [6, 8].

The human jaw/mandible has a specific motion that depends on the temporomandibular joints, which are the most sophisticated joints in the human body with three rotational and three translational degrees of freedom. These movements can be followed by observing the lower central incisor. The border movements of the incisal edge of the lower incisor describe an area or envelope called border movement area or Posselt diagram [9, 10]. Within this area, we can find all the possible positions of this structure in the mandible. Little attention has been given to the large variation of craniofacial morphology and its impact on the mandibular motion characteristics (magnitude and direction) [9, 11, 12] and in particular to the use of MAD [13]. The mandible always retrudes by postero-rotation during aperture, but depending on the craniofacial morphology, it will present different degrees of retrusion [9]. The mandible in a more retruded position in a sleep apnea patient might increase the severity of the apnea [14].

It is important to know the position of the mandible at two different moments with MAD: Starting Position (SP) and mandibular movements when the patient is asleep. A MAD is constructed with a SP placing the mandible with a determined range of advancement (50–75% of maximum protrusion) and vertical openings (2–6 mm) [15]. After the MAD is in place at the SP, we must consider the mandibular movements allowed by the appliance; lateral, anteroposterior and vertical (opening). The opening path movements have different directions according to the craniofacial morphology of the patient [9] but they are always downward and backward, therefore increasing the collapse of the upper airway [16]. All these movements can be visualized within the border movement area in order to ensure the desired advance of the mandible with the MAD. Thus, the aim of this study is to determine if there is a relationship between the different craniofacial morphologies/phenotypes and mandibular movement.

## Methods

Fifty-two dentistry students, at the Universidad Alfonso X Madrid, 29 females and 23 males, 19 to 23 years old (mean age 21.3 SD 1.7) agreed to participate in this study. Sample size was selected according to a similar previous study [9]. All subjects were asymptomatic for temporomandibular disorders, according to the Research Diagnostic Criteria/ Temporomandibular Disorders RDC/TMD, RDC /TMD [17]. The ethical review board of Universidad Alfonso X Madrid UAX approved this study UAX-2016–021. All patients had full permanent dentition up to the second molar with no previous maxillofacial surgery or TMJ symptomology.

**Fig. 1:**
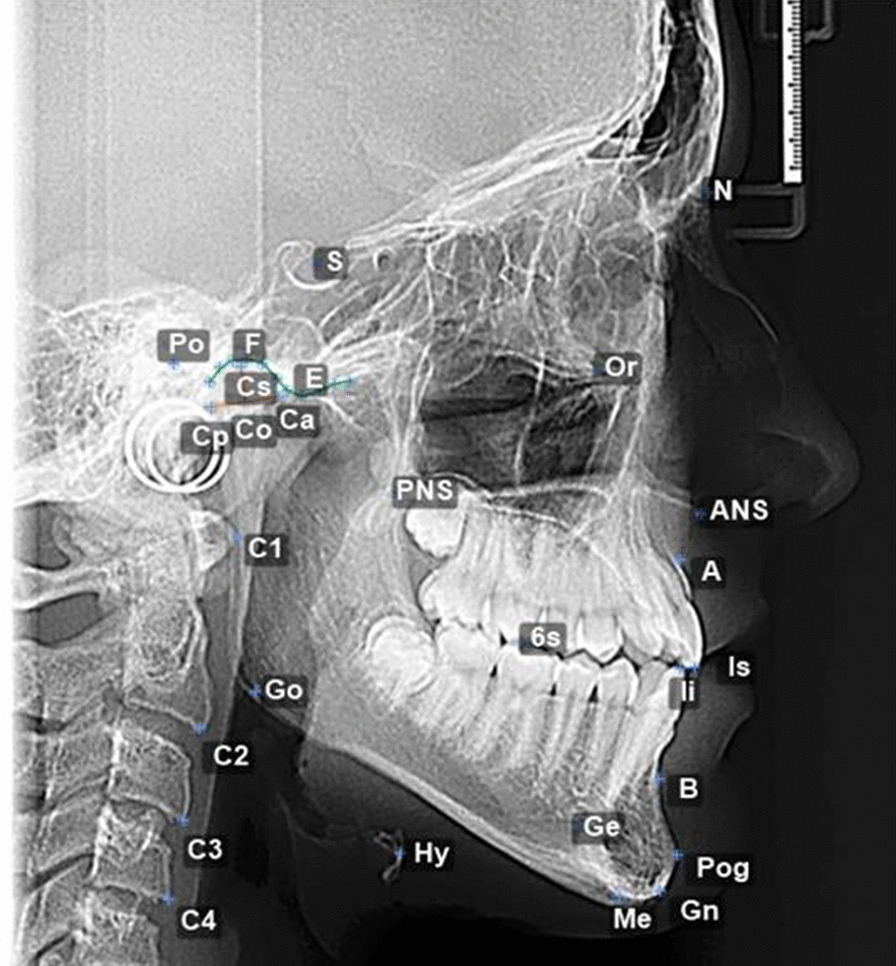
Cephalometric landmarks. Shows cephalometric radiography with landmarks as described in Table 1

Lateral cephalometric radiograph were done for each participant at the start of the study with profile and frontal extraoral photos. The Kinovea software (Kinovea, France) was used to trace landmarks on the lateral cephalograms. For each landmark, X and Y coordinates were determined with the reference at the posterior nasal spine (PNS) and using the plane anterior nasal spine (ANS) to PNS. Angles and distances were measured and placed in an excel worksheet. The landmarks with their respective definitions are listed in Table 1. Figure 1 illustrates a lateral cephalometric radiograph with landmarks. Distances and angles measured on each radiograph are listed in Table 2. Radiographs and measurement of maximum retrusión and protrusion were taken with participants’ head with Frankfurt plane parallel to the floor.

**Table 1:** Cephalometric landmarks used in the study

Landmark	Definition
6s	Mesiobuccal cusp of the upper first molar
A-point (A)	Deepest point of the maxillary base between the anterior nasal spine and the alveolar crest
Anterior Nasal Spine (ANS)	Tip of the anterior nasal spine
B-Point (B)	Deepest point in the concavity of the anterior border of the symphysis
Border movement 1 (Bm1)	Maximum retrusion point at maximum retrusion value with the George Gauge at parallel line from occlusal plane and 2 mm opening from incisal edge of upper incisor
Border movement 2 (Bm2)	Maximum protrusion point at maximum protrusion value with George Gauge at parallel line from occlusal plane and 2 mm opening from incisal edge of upper incisor
Border movement 3 (Bm3)	Rotational posterior point at 25 mm of vertical opening
Border movement 4 (Bm4)	Maximum opening point at 50 mm
Cervical vertebra (C1)	The most anterior point on the corpus of the first cervical vertebra
Cervical vertebra (C2)	The most anterior-inferior point on the corpus of the second cervical vertebra
Cervical vertebra (C3)	The most anterior-inferior point on the corpus of the third cervical vertebra
Cervical vertebra (C4)	The most anterior-inferior point on the corpus of the fourth cervical vertebra
Condyle (Co)	The center point of the condyle
Condyle posterior (Cp)	The most posterior posterior point of the condyle
Condyle superior (Cs)	The most posterior superior point of the condyle
Condyle anterior (Ca)	The most anterior superior point of the condyle
Eminence (E)	The most inferior point of the articular eminence
Fossa (F)	The most superior point of the glenoid fossa
Geni apophyse (Ge)	The most posterior point of the apophyse geni
Gnathion(Gn)	Midpoint between the most anterior and inferior point on the bony chin
Gonion (Go)	The most convex point where the posterior and inferior curves of the ascending ramus meet each other
Incisor superior (IS)	The most inferior anterior point of the incisal edge of the maxillary incisor
Incisor inferior (II)	The most superior anterior point of the incisal edge of the mandibular incisor
Mandibular Incisor Edge (II)	Tip of the mandibular central incisor
Maxillary Incisor Edge (IS)	Tip of the maxillary central incisor
Menton (Me)	Most inferior point of the symphysis
Nasion (N)	Most anterior superior point at the intersection of the nasal bone and the nasofrontal suture in the midsagittal plane
Orbitale (Or)	Most inferior point of outer border of the orbital cavity
Pogonion (Pog)	Most anterior point on the midsagittal symphysis
Porion (Po)	Most superior point of the external auditory canal
Posterior Nasal Spine (PNS)	Tip of the posterior spine of the palatine bone
Retrusion opening point	
5 mm (R5)	Point on line parallel to maxillary occlusal plane at 5 mm and crossing arch with center on Co and radius on II
Retrusion opening point	
10 mm (R10)	Point on line parallel to maxillary occlusal plane at 10 mm and crossing arch with center on Co and radius on II
Sella (S)	Center of sella turcica

Cephalometric landmarks used in the study are shown in the figure.

**Table 2:** Measurements

Measurement	Definition
Angles	
SNNA	Angle formed by the planes S–N and N-A
SNNB	Angle formed by the planes S–N and N-B
NANB	Angle formed by the planes N-A and N-B
SNGoMe	Angle formed by the planes S–N and Go-Me
SNSGe	Angle formed by the planes S–N and S-Ge
SNSGn	Angle formed by the planes S–N and S-Gn
Vertical distances	
NENA	Distance from N to ENA
ENAMe	Distance from ENA to Me
NMe	Distance from N to Me
SGo	Distance from S to Go
ENPS	Distance from S to ENP
ENPGo	Distance from ENP to Go
Horizontal distances	
CoA	Distance from Co to A
CoGn	Distance from Co to Gn
CoGo	Distance from Co to Go
GoGn	Distance from Go to Gn
GoMe	Distance from Go to Me
Especial measurements	
Disoccluding angle 5 mm	Angle formed by parallel line to occlusal maxillary plane crossing point II and line from II to R5
Disoccluding angle10 mm	Angle formed by parallel line to occlusal maxillary plane crossing point II and line from II to R10

Detailed description of the measurements used in the study.

**Fig. 2:**
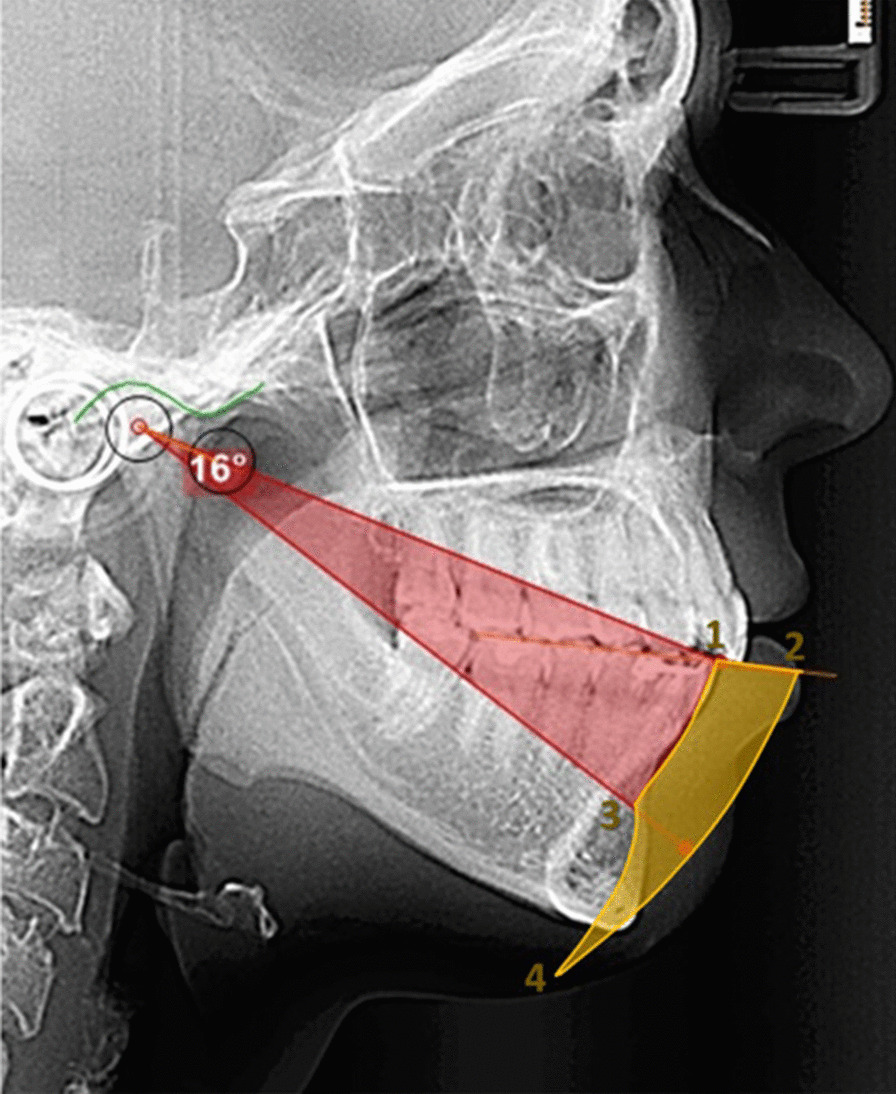
Construction of border movement area. The protrusion upper border was determined on the radiograph using the measurement maximum retrusion (point 1) and maximum protrusion (point 2). The curved posterior border in the first phase of the condyle rotation up to 25 mm opening (red area of arch) (points 1–3), and then rotation and translation up to 50 mm opening (points 3–4). The anterior curve of the diagram an arch passing by points 2 and 4 (Fig. 2) with radius at the condyle at maximum advancement. The final simplified border movement area is shown in yellow

When taking measurements patients were asked to sit straight in the dental chair. The absolute range of maximal mandibular protrusion and retrusion was measured (in mm) using the George Gauge (Great Lakes Orthodontics, Ltd., New York, USA) with a 2 mm interincisal vertical opening bite fork [13]. The principal investigator asked the patient to protrude and retrude for three times and took measurements of the maximum protrusion and maximum retrusion (Fig. 2). A simplified kinematic border movement model of the mandible in the sagittal plane was used to determine the Posselt diagram. Using the cephalometric radiograph a simplification of the Posselt diagram was calculated [18].

**Fig. 3:**
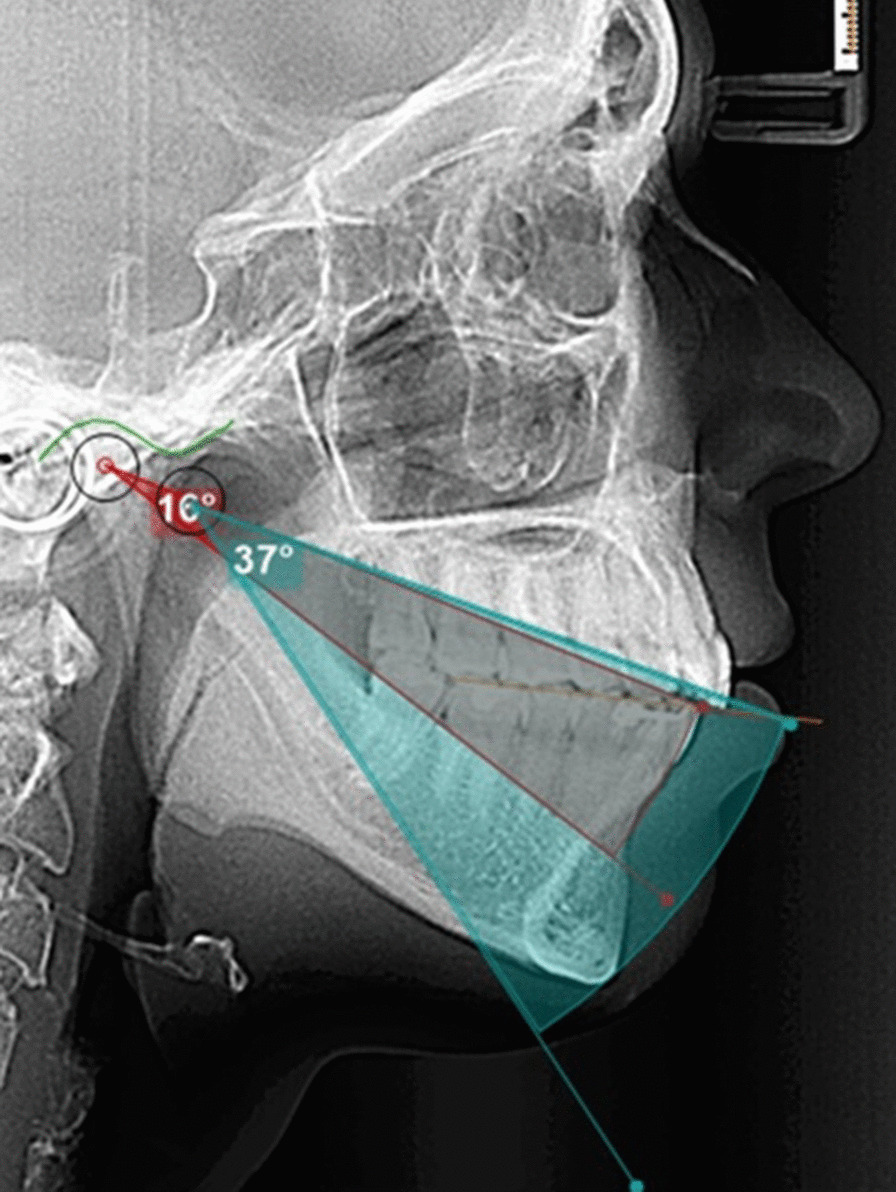
Condyle position at maximum retrusion and advancement. The condyle position at maximum advancement (blue arch) and retrusion (red arch) calculated considering the initial position of it in the radiograph and the morphology of the glenoid fossa

The protrusion upper border was determined on the radiograph with the maximum retrusion (Fig. 2 point 1) and maximum protrusion (Fig. 2 point 2) measurements taken with the George Gauge. Once the position of the incisors is found, the condyle position at maximum advancement and retrusion is calculated considering its initial position in the radiograph and the morphology of the glenoid fossa (Fig. 3).

The next step was to calculate the curved posterior border in the first phase of the condyle rotation up to 25 mm opening (Fig. 2 points 1–3) [10]. The rotational movement of the mandible in its hinge in the center of the condyles is represented as an arch. The center of the arch is located in the condyle (landmark Co) and having the curve pass through the lower incisor through the line that connects the condyle and the lower incisor (Co-II).

Once landmark 3 (Fig. 2 point 3) is reached, the condyle moves through the glenoid fossa until the lower central incisors reach the maximum opening of 50 mm (Fig. 2 point 4).

The anterior curve of the diagram was drawn by an arch passing by points 2 and 4 (Fig. 2) with the radius at the condyle at maximum advancement. Figure 2 shows the resulting Posselt diagram where the shadowed area represents the region where the central lower incisors can be placed.

**Fig. 4:**
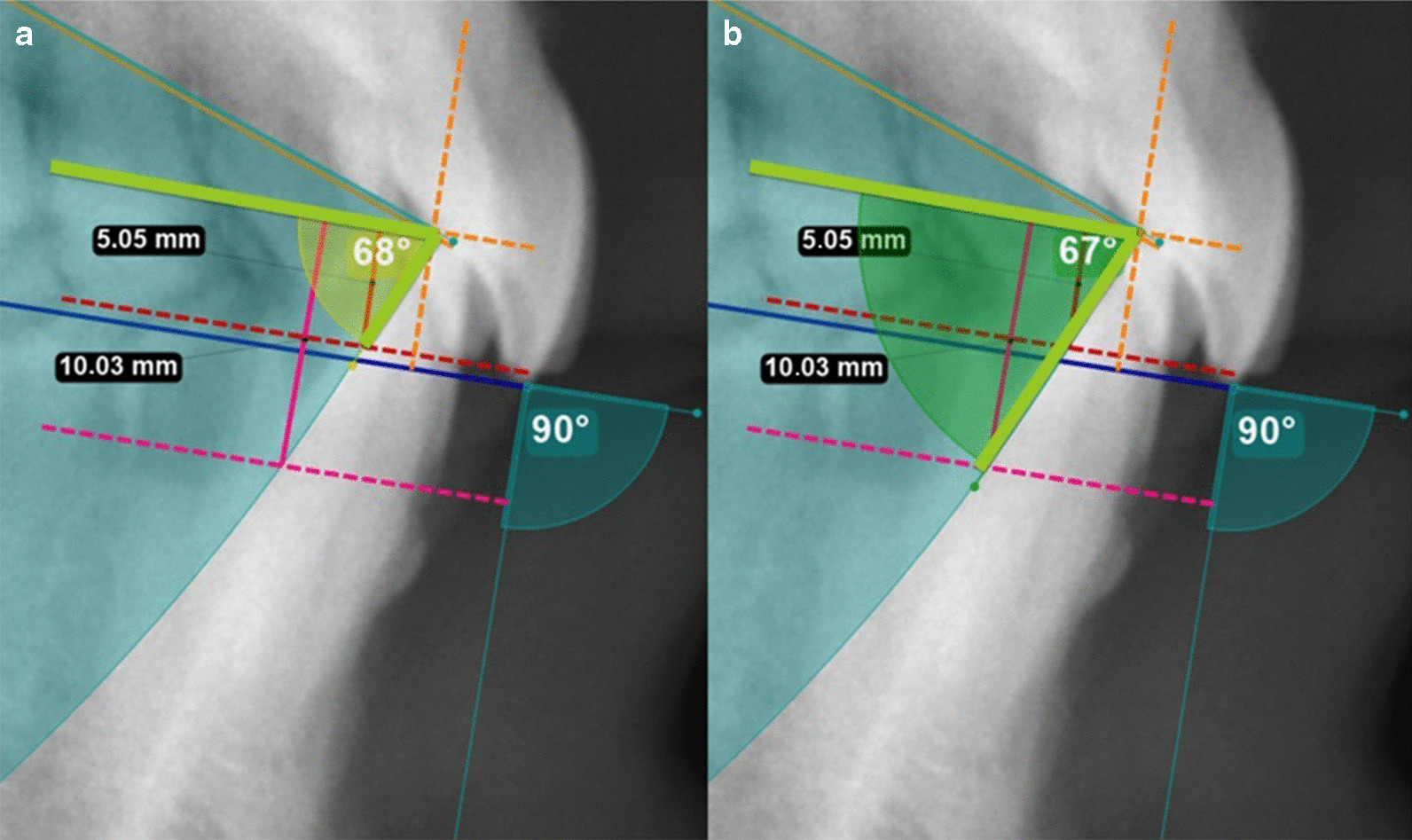
The disocclusion angle. The disocclusion angle with vertex at the border of the lower central incisors, one side parallel to the occlusal plane (green line) and the other to a point crossing the rotational curve (blue arch) of the mandible at 5 mm opening (orange dotted line) (**a**). The same was done for the 10 mm opening (pink dotted line) (**b**)

**Fig. 5:**
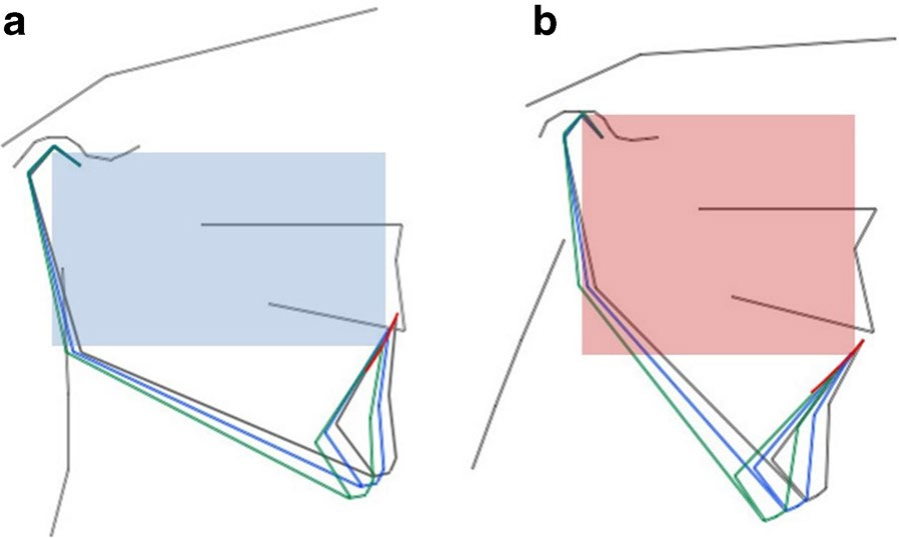
Disoccluding angle. The low angle horizontal pattern (blue area) (**a**) in a patient with higher vertical anterior and shorter anteroposterior face which describes a more vertical opening pattern and high angle vertical pattern (red area) (**b**) in a patient with a more horizontal path due to a shorter vertical anterior and longer anteroposterior face

Once the diagrams were obtained, the disocclusion angle was calculated. (Fig. 4) The disocclusion angle has its vertex at the border of the lower central incisors and one side is parallel to the occlusal plane. The other side is formed from the border of the central lower incisors to a point that crosses the rotational curve of the mandible at the 5 mm opening. The same was done for the 10 mm opening. The disocclusion angle allows us to compare/obtain the differences in the direction of the mandibular movement between subjects at the curved posterior border in the first phase of condyle rotation of the Posselt diagram (Fig. 5).

To calculate reliability and measurement error of the structures and measurements obtained, ten radiographs were randomly selected and landmarked three times leaving one week in between trials. Interclass Correlation Coefficient was used to calculate the reliability as well as the measurement error for each landmark in each coordinate (Table 3). The statistical analysis included descriptive statistics, ANOVA and paired t-test using SPSS (version 24, IBM, Armonk, NY, USA). Paired sample test and Pearson correlation were used to compare findings with respect to each disocclusion angle of 5 and 10 mm (more significant results shown in Table 4). Descriptive Statistics and Multiple Comparisons analysis was used to verify difference between the three different groups (hypodivergent, normodivergent and hyperdivergent).(Table 5).

**Table 3:** Mean measurement error of cephalometric landmarks

Cephalometric landmarks	N	Mean	SD	Mean	SD
Descriptive statistics					
PNS	10x	0.04	0.02y	0.04	0.02
N	10x	0.69	0.41y	0.60	0.33
S	10x	0.63	0.52y	0.70	0.54
Po	10x	0.82	0.40y	0.60	0.44
Or	10x	1.40	1.10y	0.84	0.25
ANS	10x	0.92	0.53y	0.65	0.38
A	10x	0.92	0.55y	1.18	1.17
IS	10x	0.58	0.41y	0.52	0.33
II	10x	0.73	0.60y	0.43	0.31
B	10x	0.58	0.26y	1.18	0.73
Pog	10x	0.61	0.40y	0.86	0.46
Gn	10x	0.75	0.44y	0.53	0.28
Me	10x	1.94	1.04y	0.55	0.35
Ge	10x	0.55	0.48y	1.24	0.86
Go	10x	1.30	1.35y	1.00	0.65
C	10x	0.72	0.46y	0.50	0.48
Cp	10x	0.82	0.77y	0.68	0.62
Cs	10x	0.68	0.57y	0.52	0.36
Ca	10x	0.67	0.45y	0.63	0.45
C1	10x	0.46	0.38y	0.96	0.56
C2	10x	0.84	0.55y	0.61	0.53
C3	10x	0.68	0.56y	0.55	0.37
C4	10x	0.63	0.50y	0.55	0.39
Fosa	10x	0.77	0.37y	0.40	0.14
E	10x	0.79	0.28y	0.59	0.35

Descriptive statistics of the reliability of the cephalometric landmarks in both the x and y axis, showing the mean and the standard deviation.

**Table 4:** Descriptive statistics and paired samples correlations

Cephalometric measurements	Descriptive statistics		Paired samples correlations		
	Mean	SD	Angle	Correlation	Significance
Vertical measurements					
*Angles*					
Or-Po > IS-6Ms	10.01º	3.97	angle5	0.55	0.000**
			angle10	0.54	0.000**
S–N > IS-6Ms	0.53º	6.16	angle5	0.23	0.088
			angle10	0.24	0.078
S–N > Go-Me	15.74º	8.02	angle5	− 0.06	0.659
			angle10	− 0.06	0.666
Distances S-Go	85.56º	10.77	angle5	− 0.19	0.175
			angle10	− 0.16	0.240
ENP-S	52.11º	3.99	angle5	− 0.16	0.247
			angle10	− 0.13	0.337
ENP-Go	50.03º	7.18	angle5	0.00	0.999
			angle10	0.02	0.866
N-Me	133.14º	11.25	angle5	− 0.10	0.450
			angle10	− 0.08	0.572
N-ENA	59.43º	5.16	angle5	− 0.01	0.942
			angle10	0.005	0.971
ENA-Me	74.55º	8.59	angle5	− 0.11	0.415
			angle10	− 0.09	0.525
Horizontal measurements					
*Angles*					
S–N > N-A	79.08º	4.88	angle5	0.04	0.738
			angle10	0.06	0.671
S–N > N-B	77.28º	4.48	angle5	− 0.22	0.108
			angle10	− 0.20	0.144
N-A > N-B	1.80º	2.93	angle5	0.42	0.002*
			angle10	0.41	0.002*
Distances N-S	79.27º	6.04	angle5	− 0.07	0.623
			angle10	− 0.04	0.747
ENP-A	54.01º	5.23	angle5	− 0.02	0.837
			angle10	0.002	0.988
Go-Gn	86.38º	9.28	angle5	− 0.10	0.467
			angle10	− 0.07	0.621

*Correlation is significant at the 0.05 level (2-tailed)

**Correlation is significant at the 0.01 level (2-tailed)

Table showing the descriptive statistics and the paired samples correlations with the more relevant vertical and horizontal measurements for both the angle at 5 mm. and the angle at 10 mm. The correlations and the significance are shown.

**Table 5:** Descriptive statistics and multiple comparisons

Cephalometric measurements		Mean	SD
Descriptive statistics			
DMi > IS-6Ms	Horizontal	22.05º	1.65
	Normal	18.83º	0.82
	Vertical	14.69º	1.56
Or-Po > IS-6Ms	Horizontal	6.92º	2.37
	Normal	9.46º	3.12
	Vertical	12.60º	4.21
N-A > N-B	Horizontal	− 0.60º	3.19
	Normal	2.06º	2.65
	Vertical	2.93º	2.36
Disocclusion angle 5	Horizontal	66.42º	1.57
	Normal	69.68º	0.78
	Vertical	73.82º	1.59

Dependent Variable:Bonferroni	Value				
*Multiple post hoc tests*					
Co-II > IS-6Ms	Horizontal	Normal	3.22º_*_	0.47	0.00**
		Vertical	7.36º_*_	0.50	0.00**
	Normal	Horizontal	− 3.22º_*_	0.47	0.00**
		Vertical	4.14º_*_	0.41	0.00**
	Vertical	Horizontal	− 7.36º_*_	0.50	0.00**
		Normal	− 4.14º_*_	0.41	0.00**
Or-Po > IS-6Ms	Horizontal	Normal	− 2.53º	1.25	0.14
		Vertical	− 5.68º*	1.30	0.00**
	Normal	Horizontal	2.53º	1.25	0.14
		Vertical	− 3.14º_*_	1.07	0.01*
	Vertical	Horizontal	5.68º_*_	1.30	0.00**
		Normal	3.14º_*_	1.07	0.01*
N-A > N-B	Horizontal	Normal	− 2.67º_*_	0.98	0.02*
		Vertical	− 3.54º_*_	1.02	0.00**
	Normal	Horizontal	2.67º_*_	0.98	0.02*
		Vertical	− 0.87º	0.84	0.91
	Vertical	Horizontal	3.54º_*_	1.02	0.00**
		Normal	0.87º	0.84	0.91

*Correlation is significant at the 0.05 level (2-tailed)

**Correlation is significant at the 0.01 level (2-tailed)

Descriptive statistics and multiple comparisons for the three opening patterns horizontal (hypodivergent), normal (normodivergent) and vertical (hyperdivergent) for the more relevant cephalometric measurements. Descriptive statistics include mean and standard deviation and multiple comparisons shows Dependent Variable Bonferoni with significance.

## Results

Reliability of cephalometric landmarks was measured on 10 random patients for three times for both x and y axis (Table 3). All landmarks presented excellent reliability values with the lowest landmark being 0.97 (CI 95% 0.94; 0.99) in the A point y-axis. It is worth noting the landmark PNS presented very low reliability but when reviewing the measurement error, these were on average 0.04 mm in both axes. The highest measurement error was found in landmark Me with 1.94 mm $\pm$ 1.05 in the x-axis. The majority of landmarks had an average of < 1 mm error in all coordinates.

Once reliability was determined, all the cephalograms were landmarked and measured. The cephalometric analysis principal vertical and horizontal measurements are shown in Table 4, and the Descriptive Statistics and Multiple Comparisons analysis between the three different groups (hypodivergent, normodivergent and hyperdivergent) in Table 5.

The range of the mandibular disoccluding angle or opening angle was determined for the two degrees of vertical openings at 5 and 10 mm. The disoccluding angle showed a greater interindividual variability from 63.15 to 77.08 with a mean value of 70.42 and standard deviation 3.06 for the angle at 5 mm and for 61.65 to 75.72 with a mean value of 68.90 and standard deviation 3.07 for the angle at 10 mm. The amount of retrusion for a 63º angle with an opening of 5 mm is 2.55 mm and for an angle of 77º is 1.15 mm. The same happens when the opening is 10 mm where the retrusion with an angle of 75º is 2.68 mm and with 61º is 5.54 mm. The opening angle was similar (*p* > 0.05) between males (70.04) and females (70.71) for the angle at 5 mm and males (68.55) and females (69.16) for the angle at 10 mm. The sample was grouped based on their measurements into hypodivergent (disoccluding angle < 68; 10 subjects), normodivergent (68 ≤ and < 71; 25 subjects) and hyperdivergent (≥ 71; 17 subjects).

The value of normodivergent, from 68 to 71 for the disoccluding angle, was taken and a retrusion of 2 mm (1.80–2.2 mm) at a vertical increase of 5 mm was considered normal. The disoccluding angle showed a high significant correlation (*p* < 0.001) with ANB angle, Frankfort-Maxillary occlusal angle and Bmi-Maxillary occlusal angle. It was found that shorter mandibles and condyles in a higher position had smaller angle values and therefore a greater horizontal opening pattern. (Table 5).

Differences of facial morphologies/phenotypes were evident when analyzing the individual opening angle of the high horizontal pattern and the low vertical pattern. (Fig. 5).

## Discussion

The physiological position of the mandible during sleep is slightly opened (1–5) mm and in patients with OSA is opened more than 5 mm and can reach up to 10 or 15 mm [19, 20]. The opening of the mandible induces mandibular retrusion, which is associated with an increase in collapsibility of the upper airway [21] and the reduction of the efficacy of the MAD [22]. Following this though, this study wanted to analyze the different opening movement paths and their impact on the mandibular position according to the craniofacial morphology of the patient.

During normal sleep, the mouth is in a position known as the mandibular rest position or freeway space. The freeway space is described as the space between the maxillary and mandibular occlusal surfaces when the mandible is in the rest position and should be 1–5 mm [23], an opening of up to 5 mm for 88.9% of total sleep time [19]. Increasing oscillating lowering movements of the mandible in response to the airway collapse during obstructive apnea have been described [24–26]. In patients with OSA, the mouth opening is greater than 5 mm for 69.3% of total sleep time [26]. A common pattern characterized by a gradual opening followed by a quick closure of the mouth, generally after an arousal, has been described in normal patients and patients with OSA [19, 24, 26]. For this reason the disoccluding angle of 5 mm that measures the normal opening in healthy patients and 10 mm that measures the opening in patients with OSA were obtained. The mandibular position and related structures are influenced by and participate in patency of the pharynx and the complex mechanisms that lead to obstruction of the upper airway [16]. Mandible opening during sleep causes mandibular posterior rotation [27] and is associated with a reduced cross-sectional area of the lumen [16], reduced mechanical efficiency of the pharyngeal dilator muscles [24] and increased resistance and collapsibility of the upper airway [14, 21, 28]. All of which may contribute to sleep-related breathing abnormalities.

The findings of the present study show/suggest that the slope or angle of the mandibular movements are related to the craniofacial morphology with higher vertical anterior and shorter anteroposterior faces with a more horizontal path of mandibular movements than shorter vertical anterior and longer anteroposterior subjects who have a more vertical path (Fig. 5). For an opening of 5 mm with an angle of 77º, the mandible retrudes 1.15 mm and with an angle of 63º is 2.55 mm. This is double for the same amount of opening at 5 mm. A similar scenario is present at the 10 mm opening where the retrusion at an angle of 75º is of 2.68 mm and at 61º is 5.54 mm which is close to a 3 mm difference.

Our results are similar to L’Estrange et al. [29] where they found a smaller effect on the oropharynx in subjects who had a reduced lower facial height. They found that in these cases, maximal mandibular protrusion had a minimal increase of vertical opening and the mandibular symphysis was further forward in relation to the posterior wall of the pharynx. The path of the mandible during aperture started in a position with the lower incisor much further forward and much closer to the cranium than in other subjects. Therefore, the mandible has a longer path to travel before it reaches the point where the airway begins to occlude. Subjects with higher vertical anterior faces and shorter anteroposteriors have an opening path beginning further backwards and downwards. As found in our study, any increase in the vertical dimension would quickly retrude the mandible [29].

**Fig. 6:**
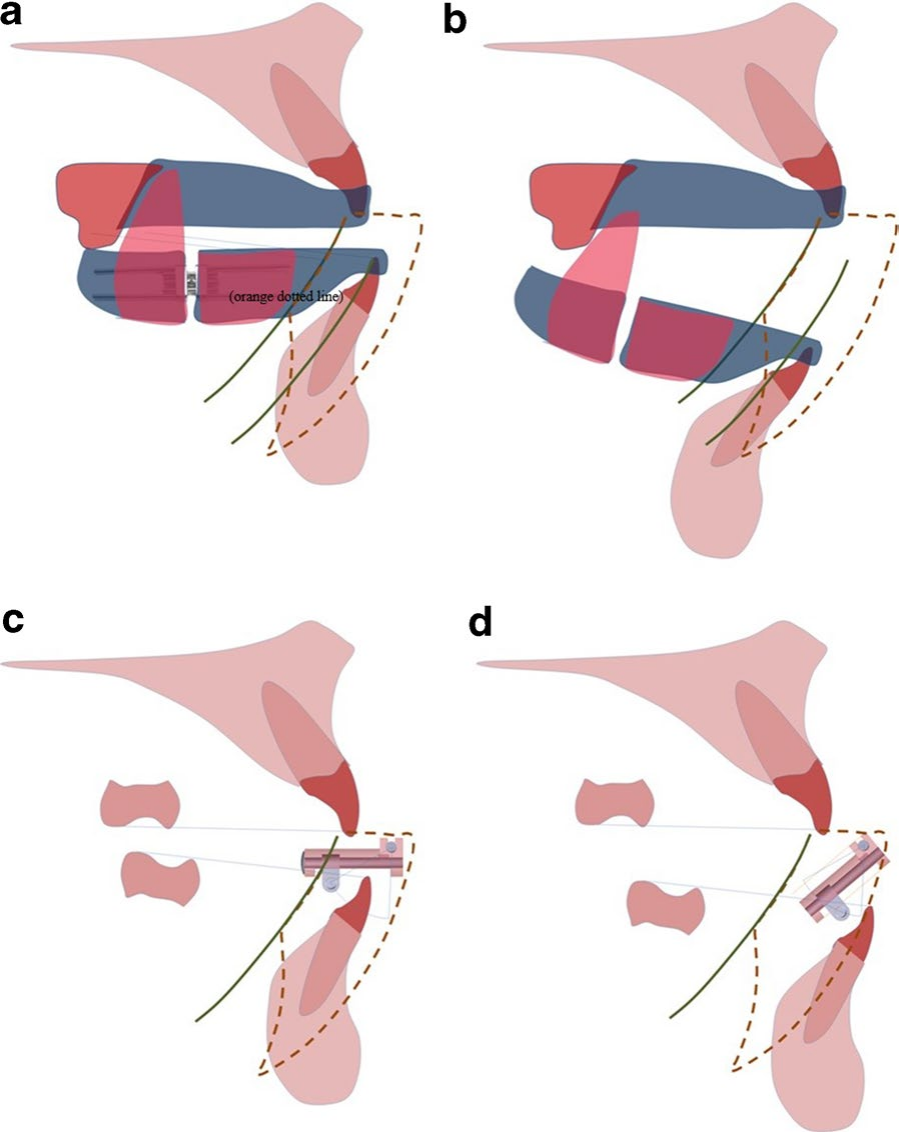
MAD Starting position and direction of jaw opening. This figure shows the mandible in the starting position when the appliance is in place (**a**, **c**) and the direction of the jaw opening direction allowed by the MAD design (**b**, **d**), for 2 different MAD designs. **a**, **b** examples of MAD with lateral wings, and **c**, **d** MAD with anterior rod

MADs place the mandible in a determined anteroposterior and vertical position to improve the upper airway cross-sectional area, due to a combination of both their effect of the protrusion of the mandible, and for their capability to stabilize the mandible [30–32] (Fig. 6). There are different MADs available in which control of the mandibular position with respect to the potential of mouth opening, especially in the supine position, depends on the MAD design [33]. A constantly larger mouth opening during sleep will reduce the efficacy of this treatment [8, 22, 28]. Two-piece appliance designs, which allow uncontrolled opening of the mandible, have shown a lower response rate in positional OSA [34], while appliances with limitation of mandibular opening are more effective in decreasing AHI [35]. Therefore, as shown in recent studies [18], the devices should incorporate vertical control in the design to not allow the jaw to move backwards at any time while opening the mouth, and as suggested by our study, consider the anatomic and kinematic of the mandible. Our results enhance recent studies that have identified the importance of phenotypic characteristics on treatment response, and suggest the relevance of MAD design features considering the kinematic behavior of the mandible as part of a personalized approach to treatment [18, 30].

Limitations of this study are the inclusion of young adult population and not OSA patients. The use of young adult population allows us to determine normal range of craniofacial morphologies and their movements that can be applied in future studies with OSA patients. Extrapolation to an OSA population can only be valid if these results are confirmed in a, typically older, more obese, OSA population compared to the young, healthy study population used in the current study. Another limitation is the use of lateral cephalometric radiograph for the analysis of mandibular movements. The radiograph was taken with the patient awake and in an upright position. The range of motion and the position of the mandible may be altered when the patient is asleep and should be consider when analyzing the changes in upper airway if measured with lateral cephalometric radiograph.

## Conclusions

The slope or angle of the mandibular movements are related to the craniofacial morphology with higher vertical anterior and shorter anteroposterior faces having a more horizontal path of mandibular movement than shorter vertical anterior and longer anteroposterior subjects who have a more vertical path.

The subjects with shorter anteroposterior and higher vertical anterior faces have low opening angles and could be described as beginning further downwards and backwards on the opening path.

Future research in an OSA population is needed.

## Data Availability

The datasets used and/or analyzed during the current study are available from the corresponding author on reasonable request.

## Abbreviations

AHI: Apnea Hypopnea Index
CPAP: Continuous positive airway pressure
MAD: Mandibular advancement device
OSA: Obstructive sleep apnea
SP: Starting position
